# Supplementary material for: Burnout Syndrome among Otorhinolaryngologists during the COVID-19 Pandemic
Source: Medicina (Kaunas). 2022 Aug 12;58(8):1089. doi: 10.3390/medicina58081089 (PMC9415072; doi:10.3390/medicina58081089)
Supplement: Supplementary file 1 [file medicina-58-01089-s001.zip › medicina-1779017-Table S1.pdf]

Table S1. The distribution of MBI results in both groups.

Subscale		ITEM	Distribution On Five Point Likert Scale*									
			Strongly disagree, n[2] (%)		Disagree, n[2] (%)		Neither agree nor disagree, n[2] (%)		Agree, n[2] (%)		Strongly agree, n[2] (%)	
			ORL[1] group	Control group	ORL[1] group	Control group	ORL[1] group	Control group	ORL[1] group	Control group	ORL[1] group	Control group
Emotional Exhaustion	1	I feel emotionally drained from my work	6 (7.5)	9 (30.0)	20 (25.0)	19 (63.4)	24 (30.0)	1 (3.3)	28 (35.0)	1 (3.3)	2 (2.5)	0 (0.0)
	2	I feel used up at the end of the workday	5 (6.2)	13 (43.4)	30 (37.5)	15 (50.0)	23 (28.8)	1 (3.3)	21 (26.3)	1 (3.3)	1 (1.2)	0 (0.0)
	3	I feel fatigued when I get up in the morning and have to face another day on the job	5 (6.2)	8 (26.7)	25 (31.3)	15 (50.0)	29 (36.3)	7 (23.3)	20 (25.0)	0 (0.0)	1 (1.2)	0 (0.0)
	6	Working with people all day is really a strain for me	5 (6.2)	9 (30.0)	30 (37.5)	13 (43.4)	23 (28.8)	7 (23.3)	21 (26.3)	1 (3.3)	1 (1.2)	0 (0.0)
	8	I feel burned out from my work	19 (23.8)	7 (23.3)	32 (40.0)	19 (63.4)	21 (26.3)	3 (10.0)	7 (8.7)	1 (3.3)	1 (1.2)	0 (0.0)
	13	I feel frustrated by my job	10 (12.5)	8 (26.6)	26 (32.5)	19 (63.4)	32 (40.0)	3 (10)	12 (15.0)	0 (0.0)	0 (0.0)	0 (0.0)
	14	I feel I'm working too hard on my job	18 (22.5)	7 (23.3)	35 (43.7)	14 (46.7)	17 (21.3)	7 (23.3)	9 (11.3)	2 (6.7)	1 (1.2)	0 (0.0)
	16	Working with people directly puts too much stress on me	18 (22.5)	23 (76.7)	35 (43.7)	6 (20.0)	18 (22.5)	1 (3.3)	9 (11.3)	0 (0.0)	0 (0.0)	0 (0.0)
	20	I feel as if I'm at my wits' end	9 (11.3)	9 (30.0)	17 (21.2)	19 (63.4)	22 (27.5)	1 (3.3)	24 (30.0)	1 (3.3)	8 (10.0)	0 (0.0)
Depersonalisation scale	5	I feel I treat some patients/clients as if they were impersonal objects	19 (23.8)	15 (50.0)	37 (46.3)	7 (23.3)	14 (17.5)	8 (26.7)	8 (10.0)	0 (0.0)	2 (2.4)	0 (0.0)
	10	I've become more callous toward people since I took this job	22 (27.5)	12 (40.0)	35 (43.7)	17 (56.7)	11 (13.8)	1 (3.3)	9 (11.3)	0 (0.0)	3 (3.7)	0 (0.0)
	11	I worry that this job is hardening me emotionally	24 (30.0)	16 (53.3)	44 (55.0)	13 (43.4)	9 (11.3)	0 (0.0)	3 (3.7)	0 (0.0)	0 (0.0)	1 (3.3)
	15	I don't really care what happens to some patients/clients	11 (13.8)	15 (50.0)	28 (35.0)	8 (26.7)	25 (31.2)	7 (23.3)	16 (20.0)	0 (0.0)	0 (0.0)	0 (0.0)
	22	I feel patients/clients blame me for some of their problems	1 (1.2)	6 (20.0)	7 (8.7)	6 (20.0)	17 (21.3)	11 (36.7)	49 (61.3)	6 (20.0)	6 (7.5)	1 (3.3)
Personal achievement	4	I can easily understand how my patients/clients feel about things	1 (1.2)	0 (0.0)	8 (10.0)	0 (0.0)	21 (26.4)	1 (3.3)	48 (60.0)	20 (66.7)	2 (2.4)	9 (30.0)
	7	I deal very effectively with the problems of my patients/clients	3 (3.7)	0 (0.0)	5 (6.3)	0 (0.0)	20 (25.0)	0 (0.0)	46 (57.5)	16 (53.3)	6 (7.5)	14 (46.7)
	9	I feel I'm positively influencing other people's lives through my work	6 (7.5)	0 (0.0)	20 (25.0)	0 (0.0)	33 (41.3)	1 (3.3)	18 (22.5)	19 (63.4)	3 (3.7)	10 (33.3)
	12	I feel very energetic	2 (2.4)	0 (0.0)	15 (18.8)	0 (0.0)	29 (36.3)	1 (3.3)	28 (35.0)	15 (50.0)	6 (7.5)	14 (46.7)
	17	I can easily create a relaxed atmosphere	3 (3.7)	0 (0.0)	14 (17.5)	0 (0.0)	18 (22.5)	4 (13.3)	39 (48.8)	14 (46.7)	6 (7.5)	12 (40.0)

		with my patients/clients										
	18	I feel exhilarated after working closely with my patients/clients	3 (3.7)	0 (0.0)	10 (12.5)	0 (0.0)	20 (25.0)	1 (3.3)	43 (53.8)	11 (36.7)	4 (5.0)	18 (60.0)
	19	I have accomplished many worthwhile things in this job	6 (7.5)	0 (0.0)	15 (18.8)	0 (0.0)	28 (35.0)	4 (13.3)	26 (32.5)	14 (46.7)	5 (6.2)	12 (40.0)
	21	In my work, I deal with emotional problems very calmly	6 (7.5)	0 (0.0)	20 (25.0)	0 (0.0)	33 (41.3)	1 (3.3)	18 (22.5)	11 (36.7)	3 (3.7)	18 (60.0)

[1]ORL: otorhinolaryngologists group; [2]n: number of subjects, *: the ORL group was composed of 80 subjects and the Control group of 30 subjects.
